# Hypoxia-mediated repression of pyruvate carboxylase drives immunosuppression

**DOI:** 10.1186/s13058-024-01854-1

**Published:** 2024-06-07

**Authors:** Michael F. Coleman, Eylem Kulkoyluoglu Cotul, Alexander J. Pfeil, Emily N. Devericks, Muhammad H. Safdar, Marvis Monteiro, Hao Chen, Alyssa N. Ho, Numair Attaar, Hannah M. Malian, Violet A. Kiesel, Alexis Ramos, Matthew Smith, Heena Panchal, Adam Mailloux, Dorothy Teegarden, Stephen D. Hursting, Michael K. Wendt

**Affiliations:** 1 Department of Nutrition, University of North Carolina at Chapel Hill, Chapel Hill, NC USA; 2 Department of Medicinal Chemistry and Molecular Pharmacology, Purdue University, West Lafayette, IN USA; 3 Purdue University Institute for Cancer Research, Purdue University, West Lafayette, IN USA; 4 Department of Nutrition Science, Purdue University, West Lafayette, IN USA; 5 Lineberger Comprehensive Cancer Center, University of North Carolina at Chapel Hill, Chapel Hill, NC USA; 6 Nutrition Research Institute, University of North Carolina at Chapel Hill, Kannapolis, NC USA; 7 Department of Microbiology and Immunology, University of Iowa, Iowa City, IA USA; 8 Holden Comprehensive Cancer Center, University of Iowa, Iowa City, IA USA; 9 Present Address: Department of Internal Medicine, University of Iowa, Iowa City, IA USA

## Abstract

**Background:**

Metabolic plasticity mediates breast cancer survival, growth, and immune evasion during metastasis. However, how tumor cell metabolism is influenced by and feeds back to regulate breast cancer progression are not fully understood. We identify hypoxia-mediated suppression of pyruvate carboxylase (PC), and subsequent induction of lactate production, as a metabolic regulator of immunosuppression.

**Methods:**

We used qPCR, immunoblot, and reporter assays to characterize repression of PC in hypoxic primary tumors. Steady state metabolomics were used to identify changes in metabolite pools upon PC depletion. In vivo tumor growth and metastasis assays were used to evaluate the impact of PC manipulation and pharmacologic inhibition of lactate transporters. Immunohistochemistry, flow cytometry, and global gene expression analyzes of tumor tissue were employed to characterize the impact of PC depletion on tumor immunity.

**Results:**

PC is essential for metastatic colonization of the lungs. In contrast, depletion of PC in tumor cells promotes primary tumor growth. This effect was only observed in immune competent animals, supporting the hypothesis that repression of PC can suppress anti-tumor immunity. Exploring key differences between the pulmonary and mammary environments, we demonstrate that hypoxia potently downregulated PC. In the absence of PC, tumor cells produce more lactate and undergo less oxidative phosphorylation. Inhibition of lactate metabolism was sufficient to restore T cell populations to PC-depleted mammary tumors.

**Conclusions:**

We present a dimorphic role for PC in primary mammary tumors vs. pulmonary metastases. These findings highlight a key contextual role for PC-directed lactate production as a metabolic nexus connecting hypoxia and antitumor immunity.

**Graphical abstract:**

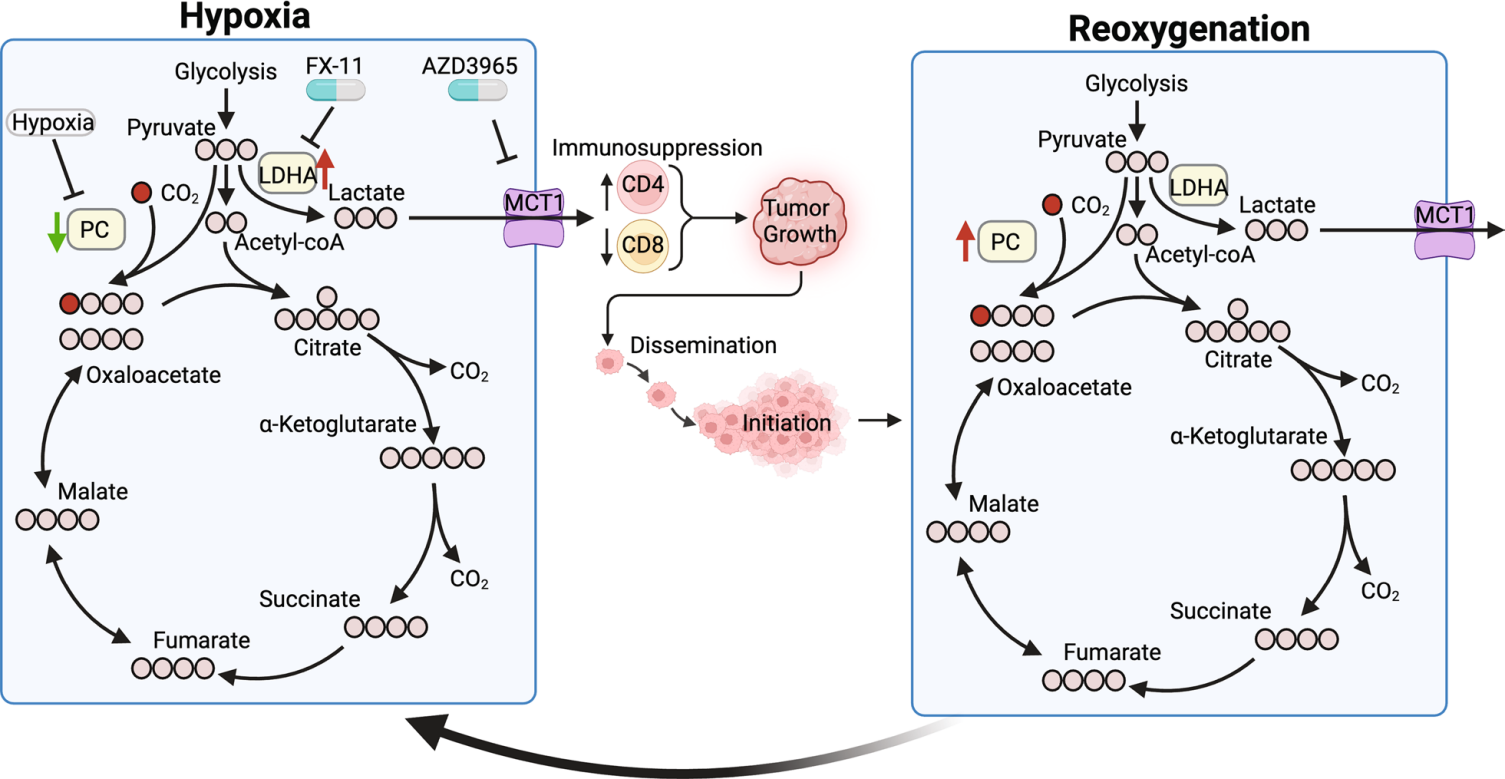

**Supplementary Information:**

The online version contains supplementary material available at 10.1186/s13058-024-01854-1.

## Background

Reprogramming metabolism and escaping immune destruction are hallmarks of cancer [1]. The interplay of cancer cells and immune cells in the tumor microenvironment (TME) is a critical determinant of cancer progression and treatment efficacy [2, 3], and is mediated in part through metabolic interactions [4–6]. Rapid cell proliferation and vasculature dysfunction frequently result in solid tumor hypoxia and subsequent reprogramming of central carbon metabolism [7]. Such hypoxia promotes local and metastatic spread via numerous mechanisms including metabolic and transcriptional reprogramming of the TME [7]. Tumoral hypoxia also alters the TME immune landscape by dampening antitumor immune responses while supporting protumor inflammatory signaling [8].

The accelerated glycolytic and biosynthetic processes in cancer cells result in the rapid depletion of essential anabolic nutrients like glucose and glutamine within the tumor microenvironment [6]. Such TME remodeling poses a hostile metabolic environment for activated cytotoxic T cells, key mediators of antitumor immunity, which compete with cancer cells for glucose and glutamine [6]. In contrast, T regulatory (Treg) cells rely more heavily on fatty acid oxidation, thus, enabling the accumulation of Treg cells in the metabolically hostile TME [9]. Lactate, produced by cancer cells at high rates, promotes both formation and activity of Treg cells to limit antitumor immunity [10] and restrict cytotoxic activity of CD8^+^ T cells [6, 11]. Indeed, high lactate levels regulate many cell types within the TME, including cancer cells, T cells, NK cells, tumor associated macrophages (TAMs), and dendritic cells to limit antitumor immunity [12–16]. Lactate secretion from tumor cells is facilitated via monocarboxylate transporters (MCTs) and is accompanied by the export of H^+^ ions acidifying the TME to drive further immunosuppression [17, 18]. Therefore, the expression and activity of these transporters play a pivotal role in immune cell function in the TME and are emerging as important drug targets for the treatment of solid tumors [16, 19–21].

Pyruvate carboxylase (PC) converts pyruvate into oxaloacetate (OAA) to replenish the tricarboxylic acid (TCA) cycle [22]. PC is essential for the development of pulmonary metastasis, with higher expression in pulmonary metastases compared to non-pulmonary tumors [23–25]. However, the relationship between PC and primary tumor growth is less well understood. Pharmacological inhibition of PC, using both immune intact and immunocompromised animals, reduces primary tumor size [23]. Conversely, we have found that shRNA-mediated depletion of PC in mammary cancer cells does not alter primary tumor size [24]. Similarly, there is no association between PC expression and breast tumor size in METABRIC data [24]. Intriguingly, PC is suppressed in TAMs from hypoxic tumors, but hypoxia alone does not reduce PC expression in macrophages [26]. Restoration of PC in TAMs promotes T cell-dependent antitumor immunity to limit tumor growth [26].

Thus, while PC clearly supports the metabolic demands of several tumor types, the relationship between PC, hypoxia, and antitumor immunity remains unclear. Hence, we sought to address the hypothesis that hypoxia-dependent suppression of PC in mammary tumors limits antitumor immunity.

## Methods

All materials used are detailed in Table 1.

**Table 1:** Materials used

Reagent or resource	Source	Identifier
*Antibodies*		
anti-CD8 antibody for IHC	Cell Signaling	Cat#98941
anti-CD4 antibody for IHC	Cell Signaling	Cat#25229
anti-PC antibody for western blot	Millipore-Sigma	Cat#HPA043922
anti-beta tubulin antibody for western blot	Developmental Studies Hybridoma Bank	Cat#E7
anti-LDH-A antibody for western blot	Cell Signaling	Cat#2012
anti-MCT-1 antibody for western blot	Abcam	Cat#ab93048
anti-HIF1a antibody for western blot	Cell Signaling	Cat#14179
anti-phospho-PDH antibody for western blot	Cell Signaling	Cat#31866
anti-AKT antibody for western blot	Cell Signaling	Cat#9272
anti-phospho-AKT antibody for western blot	Cell Signaling	Cat#4060
anti-CD45 antibody for flow cytometry	BD	Cat#564279
viability stain for flow cytometry	Invitrogen	Cat#L34961
anti-CD44 antibody for flow cytometry	BD	Cat#741227
anti-CD11c antibody for flow cytometry	BD	Cat#564986
anti-CD4 antibody for flow cytometry	BD	Cat#741913
anti-F4/80 antibody for flow cytometry	BioLegend	Cat#123124
anti-Ly6G antibody for flow cytometry	BioLegend	Cat#127633
anti-TIM3 antibody for flow cytometry	BD	Cat#747618
anti-IA/IE antibody for flow cytometry	BioLegend	Cat#107661
anti-CD19 antibody for flow cytometry	BioLegend	Cat#115531
anti-NK1.1 antibody for flow cytometry	BioLegend	Cat#108727
anti-CD11b antibody for flow cytometry	eBioscience	Cat#46-0112-82
anti-CD8a antibody for flow cytometry	BioLegend	Cat#100758
anti-CD69 antibody for flow cytometry	BioLegend	Cat#104510
anti-CD103 antibody for flow cytometry	BioLegend	Cat#121426
anti-CD3 antibody for flow cytometry	BioLegend	Cat#100216
anti-PD-1 antibody for flow cytometry	BioLegend	Cat#135224
anti-Ly6C antibody for flow cytometry	BioLegend	Cat#128055
anti-FOXP3 antibody for flow cytometry	eBioscience	Cat#17-5773-80A
*Chemicals, Peptides, and Recombinant Proteins*		
AZD3965	MedKoo Biosciences	Cat#1448671-31-5
FX-11	MedChemExpress	Cat#HY16214
Syrosingopine	Sigma-Aldrich	Cat#SML-1908
Mir05	Oroboros	Cat#60101-01
Critical Commercial Assays		
CellTiter-Glo	Promega	Cat#G7570
Lactate-Glo	Promega	Cat#J5021
Mouse tumor dissociation kit	Miltenyi Biotec	cat#130–096-730
EZNA HP Total RNA kit	Omega Bio-Tek	Cat#R6812
Verso cDNA Synthesis Kit	Thermo Scientific	Cat##AB-1453/B
Maxima SYBR Green/ROX qPCR Mastermix	Thermo Scientific	Cat#K0222
Clariom S Assay HT, mouse	Affymetrix	Cat#902972
Experimental Models: Cell Lines		
MWnt	Hursting lab	NA
E0771	Wendt lab	NA
4T1	Wendt lab	NA
D2.A1	Wendt lab	NA
MDA-MB-231	ATCC	CRM-HTB-26
Experimental Models: Organisms/Strains		
Mouse: C57BL6/J: wildtype	Jackson labs	Strain id: 000664
Mouse: BALB/c: wildtype	Charles River	Strain id: 028
Mouse: NSG: NOD.Cg-*Prkdc*^*scid*^* Il2rg*^*tm1Wjl*^/SzJ	University of North Carolina at Chapel Hill Animal Models Core	NSG
*Oligonucleotides*		
*See Table S1 for oligonucleotide primer sequences*		
Recombinant DNA		
pLV PC over expression	This manuscript	NA
pLV empty vector	This manuscript	NA
pLKO.1 scram shRNA	Dharmacon	RHS6848
psi-LVRU6H scram shRNA	Genecopoeia	CSHCTR001
pLKO.1 shPC25 shRNA	Dharmacon	RMM3981-201836951
pLKO.1 shPC28 shRNA	Dharmacon	RMM3981-201846492
psi-LVRU6H shPC-B shRNA	Genecopoeia	MSH074157
psi-LVRU6H shPC-C shRNA	Genecopoeia	MSH074157
SMARTvector inducible shPC shRNA	Dharmacon	V3SM11253-234391411
PC promotor luciferase reporter	This manuscript	NA
Software and Algorithms		
FlowJo	FlowJo	FlowJo V10.8.1
ImageJ	FIJI	FIJI V2.13.1
Graphpad Prism	Graphpad Software Inc	Graphpad Prism V9.5.1
KMPlotter	Győrffy et al.[33]	NA
GSEA	Subramanian et al., Liberzon et al., and Reimand et al.[30–32]	V4.3.2
CIBERSORTX	Newman et al.[28]	NA

## Experimental models

### Animal husbandry

Female 8–12-week old C57BL6/J, BALB/c, and NSG mice were provided ad libitum access to food and water. C57BL6/J mice used in E0771 studies were fed D12450J (Research Diets, New Brunswick, NJ). C57BL6/J mice used in M-Wnt studies were fed AIN-93G (Research Diets). BALB/c mice used in 4T1 studies were fed standard chow diet (Teklad Global 2018S, Envigo, Indianapolis, IN). NSG mice were fed standard chow diet. Mice were monitored by vivarium staff daily for signs of dehydration, pain, or distress. Only female mice were studied as male sex hormones could confound the study of TNBC.

### Lung colonization

Mice (5/group) were injected via the lateral tail vein with control and PC-depleted E0771 cells (10^6^/100 µl) and their pulmonary tumor growth was observed with bioluminescence imaging using an Advanced Molecular Imager (AMI) (Spectral Instruments, Tucson, AZ) for 28 days. Mice were subsequently euthanized, and the lungs were then removed and fixed in 10% neutral buffered formalin for 48 h, and paraffin embedded.

### Primary tumor studies

Control and PC-depleted E0771 (5 × 10^5^/50 µl) or M-Wnt (5 × 10^4^/50 µl) cells were orthotopically transplanted in PBS into the fourth mammary fat pad of C57BL6/J mice (n = 3–6/group for each experiment), or NSG mice (n = 10/group). PC promoter- luciferase reporter 4T1 cells (4T1-PC-FF) or PC overexpressing (o.e.) 4T1 cells were orthotopically transplanted (2.5 × 10^4^/50 µl) into BALB/c mice and grown for 14–21 days before euthanasia. Body weight was measured weekly and tumor volume (0.5 × length × width^2^) was measured at least twice per week. Primary tumors were weighed; half of each tumor was snap frozen in liquid nitrogen and stored at -80 °C and the other half was formalin fixed and paraffin embedded. Mice bearing doxycycline-inducible shPC tumors were randomized to receive either control or 150 µg/ml doxycycline in their drinking water.

### Tumor resection studies

Orthotopic E0771 tumors were allowed to develop until they reached 200mm^3^ in volume at which point mice the primary tumor was surgically removed. Weekly bioluminescence was used to monitor the development of lung metastasis, and lung weight was determined at euthanasia 10 weeks later.

### In vivo* AZD3965 treatment*

For animals treated with AZD3965, control and PC-depleted E0771 tumors grew for 12 days and then were randomized to begin daily treatment with AZD3965 by oral gavage (50 mg/kg).

### Cell culture models

A549, 4T1, E0771, D2.A1, metM-Wnt^lung^, and MDA-MB-231 cells were maintained in Dulbecco’s modified Eagle’s medium (DMEM) supplemented with 10% fetal bovine serum (FBS) and 1% penicillin–streptomycin. A549 cells were deleted of HIF-1a via recombinant Cas9 ribonucleoprotein (RNP) complexes formed by incubating equal amounts of each 200 mM single guide RNA (sgRNA; described below) or each control guide RNA (cgRNA, described below) with 200 mM trans-activating CRISPR RNA (tracrRNA; IDT) at 95 °C for 5 min. sgRNA/tracrRNA duplexes were then incubated at a 1:1.2 molar ratio with recombinant Cas9 protein (IDT) at room temperature for 10 min. These preparations were then transfected via electroporation. sgRNAs design IDs; Hs.Cas9.HIF1A.1.AB (CCUCACACGCAAAUAGCUGA) and Hs.Cas9.HIF1A.1.AC (ACAGUAACCAACCUCAGUGU) were used. Control guide RNAs were purchased from IDT (Alt-R® CRISPR-Cas9 Negative Control crRNA #1 and #2). Single HIF1 α knockout clones were confirmed by qRT-PCR and immunoblot. Where indicated cells were seeded into media with glucose concentrations decreased to 5.6 mM. M-Wnt cells were maintained in RPMI containing, 10% FBS, 1% penicillin-streptomycin, 11 mM glucose, and 4 mM glutamine, prior to experiments M-Wnt cells were seeded in human plasma like media (HPLM) containing 10% FBS and 1% penicillin–streptomycin.

PC was suppressed in E0771 and M-Wnt cells by lentiviral transduction for 48 h, using particles generated by transfection of HEK293T cells with psPAX2, pMD2.G, and Pcx-targeting shRNAs in pLKO.1 or psi-LVRU6H, respectively. TRC lentiviral mouse *Pcx*-targeting shRNAs (lentiviral pLKO.1 TRC cloning vector) were purchased from GE Dharmacon. The target shRNA sequences were AAAGGACAAATAGCTGAAGGG (shPC25), TTGACCTCGATGAAGTAGTGC (shPC28), and TTCTCCGAACGTGTCACGT (Scram) and were selected for using puromycin (5 µg/ml). Similarly, Pcx-targeting shRNA were purchased from Genecopoeia in a psi-LVRU6H vector. The sequences were GAGTTGGAAGAGAATTACAC (shPC-B), CCACAACTTCAACAAGCTCT (shPC-C), and GCTTCGCGCCGTAGTCTTA (Scram) and were selected for using hygromycin (100 µg/ml). Doxycycline inducible PC suppression was achieved by transducing a GE Dharmacon SMARTvector encoding Pcx-targeted shRNA (TGCAATCGAAGGCTGCGTA) into M-Wnt cells as described above and selecting with puromycin (5 µg/ml). To generate PC promotor luciferase reporter cells, the PC promotor fragment from pGL3.0-PC-575-luc [27] was subcloned into pGL4.0 expression vector at KPN1 and XHO1 sites, and transfected into cells. Stable expression of this construct was selected for using puromycin (5 µg/ml). Where indicated, these cells also stably expressed renilla luciferase driven by a CMV promoter, and selected for using hygromycin (500 µg/ml). The 4T1, E0771 and MDA-MB-231 cell lines were separated constructed to stably express CMV-driven firefly luciferase under zeocin selection to allow for luminescent imaging of metastatic growth. PC o.e. 4T1-FF cells were generated by lentiviral transduction of full-length PC CDS or an empty vector control. All cell lines were tested for mycoplasma using R&D Systems MycoProbe Mycoplasma Detection Kit or ATCC Universal Mycoplasma Detection Kit.

## Analytical methods

### RNA extraction and qPCR

RNA was isolated from PC-depleted E0771 and M-Wnt tumors by homogenization in TRIzol, followed by chloroform extraction, and isolation using EZNA HP Total RNA kit. For qPCR analyses, cells were grown at a concentration of 2 × 10^5^ cells/well in corresponding treatment media for 24 h. The next day, cells were harvested, RNA was isolated and cDNA was synthesized. Quantitative PCR was performed using Maxima SYBR Green/ROX qPCR Mastermix using a Biorad CFX Connect Real Time System (Biorad Laboratories, Inc.). Primer sequences were obtained from the Integrated DNA Technology web site. , *Rpl4, Ubc,* *Gapdh* or *18S* were used as housekeeping genes to normalize the gene expression level. The relative difference in gene expression level was calculated using the delta-delta-cycle threshold method.

### Transcriptomic analysis

Sense-strand cDNA was synthesized, fragmented, labeled, and hybridized onto a Clariom S peg plate. The GeneChip® WT PLUS Reagent Kit (Affymetrix) was used to prepare the samples. Labeled cDNA was hybridized to the plate using GeneTitan Hybridization Wash and Stain Kit for WT Arrays (Affymetrix). Quality control and differential gene expression was conducted using Transcriptome Analysis Console (TAC v.4.0.1) software (Affymetrix). Genes were considered differentially expressed if FDRq < 0.05. Transcriptomic data subset using genes from Wikipathways “oxidative phosphorylation” or “electron transport chain”, and visualized by hierarchical clustering and principle component analysis using R. Digital cytometry was performed using CIBERSORTx [28], with previously identified mouse specific immune cell signatures [29]. Differences in cell fractions were determined by t-test.

### Gene set enrichment analyses (GSEA)

Affymetrix transcriptomic profiling data was exported using TAC, and GSEA [30] was conducted using the Hallmark gene sets [31]. FDRq < 0.05 was considered significant. For M-Wnt tumors GSEA was also conducted using the Gene Ontology Biological Processes and significant enriched/suppressed gene sets (FDRq < 0.05) were then visualized by enrichment mapping [32].

METABRIC and breast TCGA mRNA data were accessed via cBioPortal and single sample ssGSEA (ssGSEA) analysis was performed on all patients using the gene set ‘GOBP_HYPOXIA_INDUCIBLE_FACTOR_1ALPHA’. Patients were then grouped into quartiles of ssGSEA scores. The PC expression from Q1 (lowest ssGSEA scores) and Q4 (highest ssGSEA scores) was visualized, and Mann–Whitney U test was performed.

### Immunotherapy response

The correlation of overall survival of patients treated with any anti-PD1, -PDL1, or -CTLA4 associations with PC expression was assessed using automatically determined expression threshold with KMPlotter[33].

### Normal versus cancer analysis

Normal and tumor expression levels of PC across multiple tissues were obtained from the Gene Expression database of Normal and Tumor tissues (GENT2) data base [34], and analyzed by Mann–Whitney U test.

### Immunohistochemistry

Rehydrated 5 µm FFPE sections underwent antigen retrieval for 1 h in a steamer using pH 6.0 citrate buffer, and then progressively blocked with H_2_O_2_ for 10 min and blocking buffer (1% BSA with 5% goat-serum, TBS pH 7.6) for 10 min. Primary antibody incubation occurred overnight at 4 °C using anti-CD4 (1:200) or anti-CD8 (1:200) antibodies in blocking buffer. Biotinylated secondary anti-rabbit and anti-mouse antibodies (Biolegend Inc. #406401 and #405303) were applied for 1 h and then incubated with ABC (Vectastain, Vector Laboratories #PK-6100) for 30 min. Finally, samples were incubated with DAB (Vector Laboratories #SK-4100) for 5 min and counterstained with hematoxylin, before being dehydrated and mounted. Visualization of the samples were performed with a light microscope (Nikon Eclipse TS100, Germany) at 20× magnification and positive staining quantification was performed by ImageJ (imagej.nih.gov/ij/download.html, MD, USA). At least 6 randomly selected fields per slide were evaluated by two microscopists one of whom was blinded to groups. The mean positive cell count for each slide was then used for analysis.

### Metabolomics analyses

E0771 cells were seeded at a density of 2 × 10^5^ cells/plate in treatment media. The next day, metabolites were extracted using acetonitrile/methanol/water and analyzed by the Metabolomics Center at the University of Illinois Urbana Champaign. Hentriacontanoic acid was added to each sample as an internal standard prior to derivatization. Metabolite profiles were acquired using an Agilent GC–MS system (Agilent 7890 gas chromatograph, an Agilent 5975 MSD, and an HP 7683B autosampler). The spectra of all chromatogram peaks were evaluated using the AMDIS 2.71 and a custom-built database with 460 unique metabolites. All known artificial peaks were identified and removed prior to data mining. To compare between samples, data were normalized to the internal standard in each chromatogram, and each sample expressed as relative to control.

### Cell viability and proliferation assays

E0771 cells were seeded at 2 × 10^3^ cells/well in a 96-well plate into low glucose media overnight, then treated with indicated AZD3965 or syrosingopine concentrations. After 24 h of AZD3965 or syrosingopine treatment, cell numbers were quantified by CellTiter-Glo. Viability was calculated relative to the untreated control. All experiment conditions had six technical repeats and experiments were repeated at least three times.

M-Wnt cells were seeded at 2.5 × 10^3^ cells/well in a 96-well plate in HPLM overnight, then treated with 25 µM FX-11. After 24 h of FX-11 treatment relative cell numbers were quantified by staining with 100 µl of MTT solution (0.5 mg/mL 3-(4,5-dimethylthiazol-2-yl)-2,5-diphenyl tetrazolium bromide in HPLM without FBS) for 90 min and solubilized in 100 µl DMSO. Cytotoxicity was calculated relative to untreated control.

### Hypoxia

Hypoxic cell culture was achieved by displacing atmospheric O_2_ in a humidified modular incubator chamber (Billups-Rothenberg, Del Mar, CA, #MIC-101) with at least 100 L of 1% O_2_, 5% CO_2_, and 94% N_2_ gas mixture and then incubating at 37 °C for 48 h. Normoxic control cells were cultured at atmospheric O_2_ at 37 °C with 5% CO_2_.

### Western blotting

Western blotting was performed using 20 µg of protein isolated in RIPA buffer, resolved on polyacrylamide gels, and transferred to 0.45 µm PVDF membrane. After blocking for 1 h in 1% BSA/TBST buffer, the membranes were incubated overnight at 4 °C with anti-PC antibody (1:500), anti-beta tubulin antibody (1:2000), anti-LDH-A (1:1000), anti-MCT-1 (1:1000), anti-HIF1ɑ (1:1000), anti-PDH (1:1000), anti-phsophoPDH (1:1000), anti-AKT (1:1000), or anti-phosphoAKT (1:1000).

### Lactate-glo assay

The Lactate-Glo assay (Promega, Wisconsin, USA) was used to detect intracellular and extracellular lactate levels in vitro and in vivo. For the in vitro analyses, E0771 and M-Wnt cells were seeded at a concentration of 5 × 10^3^ cells/well in 96-well plates overnight, then the media was exchanged for treatment media. After a 24 h incubation with MCT-1 inhibitors or FX-11, luminescence values were measured according to the manufacturer’s instructions. Ex vivo tumor lactate was determined by homogenization of 20 mg of tumor in PBS followed by centrifugation (10^4^ RCF, 4 °C, 5 min). Lactate levels were determined by Lactate-Glo assay, normalized by total protein, and expressed as relative to scram.

### Extracellular flux analysis

Seahorse Metabolic Flux Analyzer XFe96 or XFe24 instruments (Agilent Seahorse Technologies, Santa Clara, CA) were used to determine cellular oxygen consumption rate (OCR) of in vitro scram and PC-depleted M-Wnt, D2.A1, and E0771 cells. Cells were seeded into Seahorse cell culture plates at a density of 5 × 10^3^ M-Wnt cells/XFe96 or 3 × 10^4^ E0771 or 2 × 10^4^ D2.A1 cells/XFe24 well overnight. 1 h prior to analysis, cells were incubated at 37°C in assay media (serum-free RPMI-1640 media with 10mM glucose, 2mM glutamine, and 1mM pyruvate, without bicarbonate, pH 7.4) at atmospheric CO_2_. Oligomycin (1.0 $\mu$M), carbonyl cyanide-4-(trifluoromethoxy)phenylhydrazone (FCCP; 1.0 µM), and rotenone/antimycin A (0.5 µM) were injected sequentially. OCR data was normalized to total protein using a bicinchoninic acid protein assay (Thermo Fisher, Waltham, MA), and then expressed relative to control.

### High-resolution respirometry

An established substrate-uncoupler-inhibitor-titration (SUIT) protocol for high-resolution respirometry (HRR) was utilized with in vitro control and ShPC cells (SUIT-001 O2 ce-pce DOO4, [35]). Cells were re-suspended in Mir05 buffer (Oroboros Instruments, Innsbruck, Austria). 1,000,000 cells were injected into 2 mL pre-calibrated Oxygraph-2k chambers containing Mir05 (O2k, Oroboros Instruments, Innsbruck, Austria). All experiments were performed at 37 °C under constant stirring with oxygen concentrations maintained between 100 and 200 µM. Reoxygenation, as needed, was performed via addition of 5 µl of catalase (112,000U/mL dissolved in Mir05, Sigma C9322) and titration of hydrogen peroxide (50 wt. % in H_2_O, Sigma 516,813) until desired oxygen concentration was reached. Residual oxygen consumption (ROX) was measured after permeabilization with digitonin and subtracted from oxygen flux as a baseline for all respiratory states to obtain mitochondrial respiration. Specific flux was expressed as oxygen consumption per million cells ($\rho$ mol$\bullet$s^−1^$\bullet$million cells). To determine flux control ratios (FCR), respiration values for each replicate were normalized to its respective NS_E_ specific flux. DatLab Software (V7.4, Oroboros Instruments) was utilized for data acquisition and post-experimental analysis.

### Flow cytometry

Tumors were dissociated into single cells using Miltenyi Biotec tumor dissociation kit according to manufacturer’s protocol with enzyme R reduced to 20%. Leukocytes were enriched using a percol gradient, which were passed through a 70 μm filter to ensure single cells. FC receptors were blocked with TruStain FcX (1:50, Biolengend), and BD Horizon Brilliant Stain Buffer. Cell surface markers were stained in the presence of live/dead blue viability dye (1:1600) at the following dilutions 1:150 Ly6G, CD19, and NK1.1; 1:200, CD11c, CD4, F4/80, CD11b, CD8a, CD103, CD3, FOXP3, and PD1; 1:300, CD45 and Ly6C; and 1:400, CD44, IA/IE, and CD69. All samples were measured using full spectrum flowcytometry with a Cytek Aurora, and analyzed using FlowJo.

## Statistical analysis

Python 3.8.5, R 4.0.2, Graphpad Prism 9 software was used for statistical analysis. R packages used were FactoMiner 2.4, factoextra 1.0.7, gplots 3.1.1, and ggpubr 0.4.0. One- and two-way ANOVAs with Tukey’s post-hoc tests, were used where three or more groups existed and t-tests were used to compare two groups. Welch’s correction was applied where variance was not similar between groups. P-values of less than 0.05 were considered significant. Where multiple comparisons were made, p values were considered significant where FDRq < 0.05. No exclusion criteria were utilized in these studies.

## Results

### *A dichotomous role for PC in* pulmonary *versus primary tumors*

**Fig. 1:**
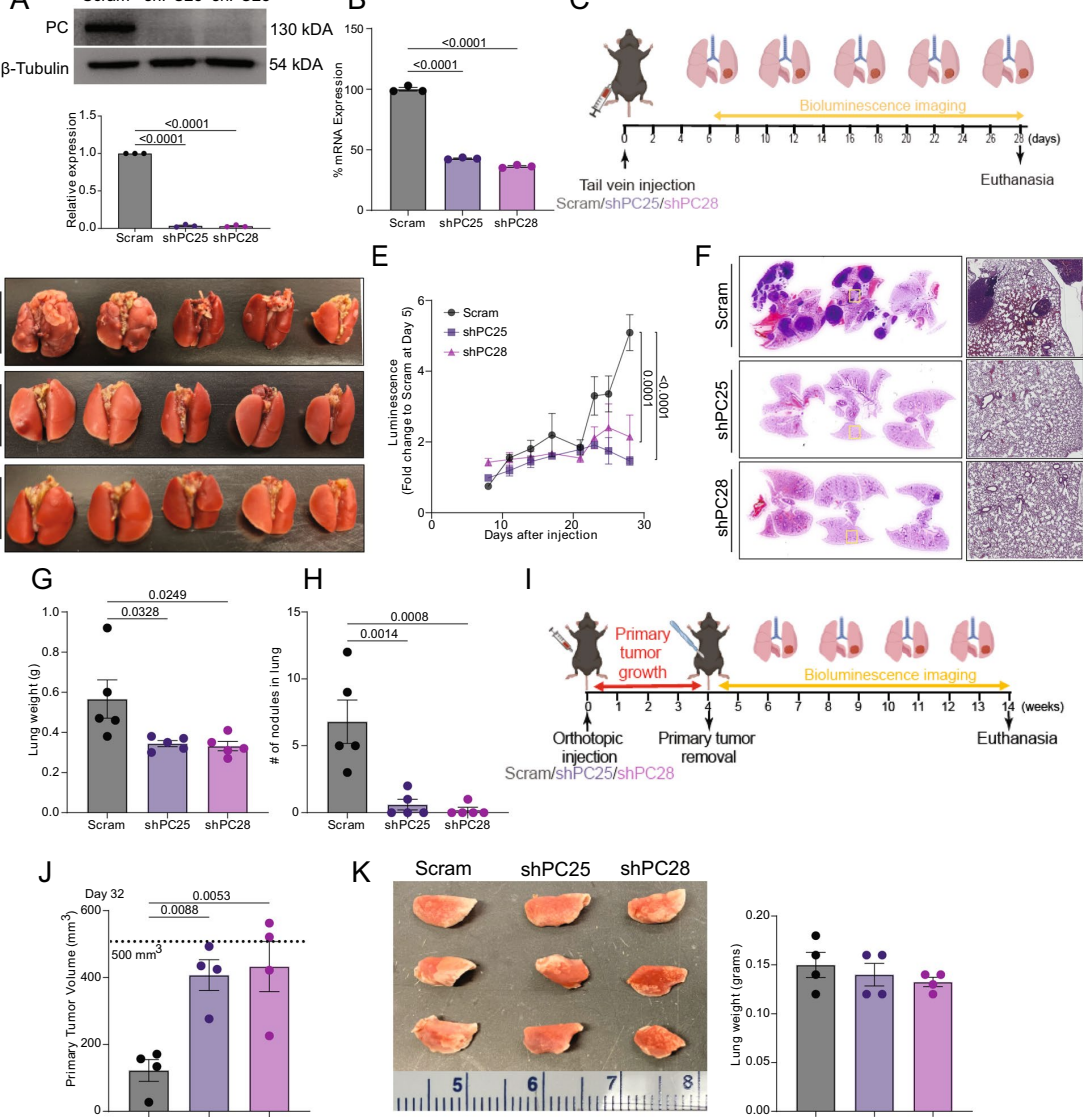
A dichotomous role for PC in pulmonary versus primary tumors. **A** Immunoblot analyses of control (scram) and PC-depleted (shPC25 and shPC28) E0771 cells probed for PC and β-tubulin. **B** Expression of PC in control and PC-depleted E0771 cells was quantified by qPCR (n = 3/group). **C** Experimental timeline of E0771 injected C57BL6/J mice. Mice were injected via the lateral tail vein with 10^6^ E0771 cells. Progression of pulmonary tumors was tracked by bioluminescence imaging every other day for 28 days. **D** Lungs from mice injected with control and PC-depleted E0771 cells. **E** Pulmonary luminescence of the 3 groups (5/group) of mice between Day 8 and 28 following tail vein inoculation. **F** H&E staining of lung histological sections (n = 3/group). **G** Lung weight and **H** pulmonary tumor nodule counts at euthanasia (5/group). **I** Experimental timeline where 5 × 10^5^ control and PC-depleted E0771 cells were orthotopically transplanted onto the mammary fat pads of C57BL6/J mice (5/group). After 4 weeks, primary tumors were removed. Progression of pulmonary metastasis was tracked by bioluminescence imaging for another 10 weeks and animals were sacrificed. **J** Primary tumor volume measurements of control and PC-depleted tumors (n = 4/group). **K** Images and weights of lungs at euthanasia (n = 4/group). Statistical significance determined by one-way ANOVA with Tukey's post-hoc test

Our previous studies utilized several BALB/c syngeneic models of breast cancer to demonstrate that PC is required for metastatic colonization of the lungs [24]. To extend these observations, we similarly conducted tail vein injection assays in C57BL/6 J mice using syngeneic E0771 mammary tumor cells with or without knockdown of PC expression (Fig. 1A–C). Consistent with our previous work, the development of pulmonary tumors following tail vein injection was attenuated in PC-depleted E0771 cells relative to scramble control (Fig. 1D–H). In addition to differences in bioluminescence, total lung weight, and gross enumeration of macroscopic lesions, histological examination of lungs indicated an absence of microscopic lesions in PC-depleted cells (Fig. 1F). To investigate the impact of PC depletion on primary tumor growth, the same scrambled control and PC-depleted cells were orthotopically transplanted into the 4th mammary fat pad (Fig. 1I). In contrast to the inhibitory effect of PC suppression on tumor growth in the lungs following tail vein injection, orthotopic.

tumors from PC-depleted E0771 cells were larger than the scrambled control (Fig. 1J). Spontaneous metastases were not found in either PC-depleted or scrambled control tumor bearing mice (Fig. 1K).

**Fig. 2:**
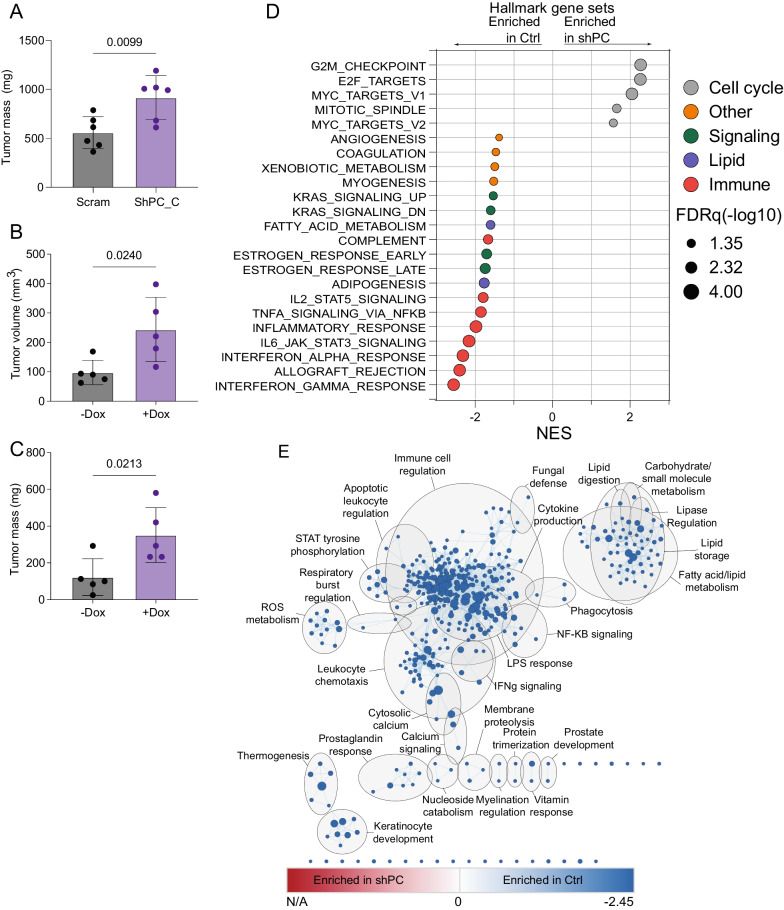
Depletion of PC increases primary tumor growth and suppresses immune related gene expression. **A** Tumor mass from M-Wnt tumors harboring control (scram) or PC-targeted (ShPC-C) constitutive shRNAs (n = 6/group). **B** Tumor volume and **C** tumor mass from M-Wnt tumors harboring PC-targeted dox inducible shRNA treated with (+Dox) or without doxycycline (- Dox) (n = 5/group). **D** Hallmark GSEA of transcriptomic profile of tumors from B-C, which were significantly enriched with FRDq < 0.05 (n = 5/group). **E** Enrichment maps of significant (FDRq < 0.05) GSEA Gene Ontology Biological processes in Ctrl and shPC tumors. (FDRq < 0.05). Node color indicates normalized enrichment score, node size indicates gene set size, line weight indicates degree of overlap, and clusters indicate minimum 50% overlap of gene sets. Statistical significance was determined by unpaired student’s t-test (**A**–**C**)

To further investigate the role of PC on orthotopic mammary tumor growth, we utilized M-Wnt cells, another C57BL/6 syngeneic tumor model. Using these cells, we established doxycycline-inducible and constitutive models of PC suppression. Orthotopic tumors from constitutive PC-depleted M-Wnt cells were larger than scrambled control tumors (Fig. 2A). Consistent with constitutive PC-depletion in E0771 and M-Wnt cells, suppression of PC by doxycycline treatment resulted in significantly larger tumors relative to non-doxycycline treated control mice following orthotopic injection of M-Wnt cells with a doxycycline-inducible shPC construct (Fig. 2B, C). To identify major pathways and processes disrupted by suppression of PC, we conducted global transcriptomics using an Affymetrix microarray followed by gene set enrichment analysis (GSEA). We found striking suppression of GSEA Hallmark gene sets related to immune signaling in the shPC group relative to control M-Wnt tumors (Fig. 2D). To identify more granular pathway alterations, we next conducted GSEA using Gene Ontology Biological Processes gene sets with enrichment mapping to minimize redundancy. We found that relative to control tumors, PC-depleted tumors had disrupted immune-related and fatty acid/lipid metabolism signaling (Fig. 2E).

We next conducted digital cytometry to identify potential immune cell populations altered by suppression of PC and identified an increase in M0 macrophages and a reduced abundance of Th1 and resting NK cells (Additional Fig. 1A–E). We confirmed these effects in orthotopic E0771 tumors by performing transcriptomic analysis followed by GSEA using Hallmark gene sets on scrambled control and PC-depleted E0771 tumors. We once again found that PC depletion in tumor cells suppressed tumor immune-related signaling relative to control tumors (Additional Fig. 2).

**Fig. 3:**
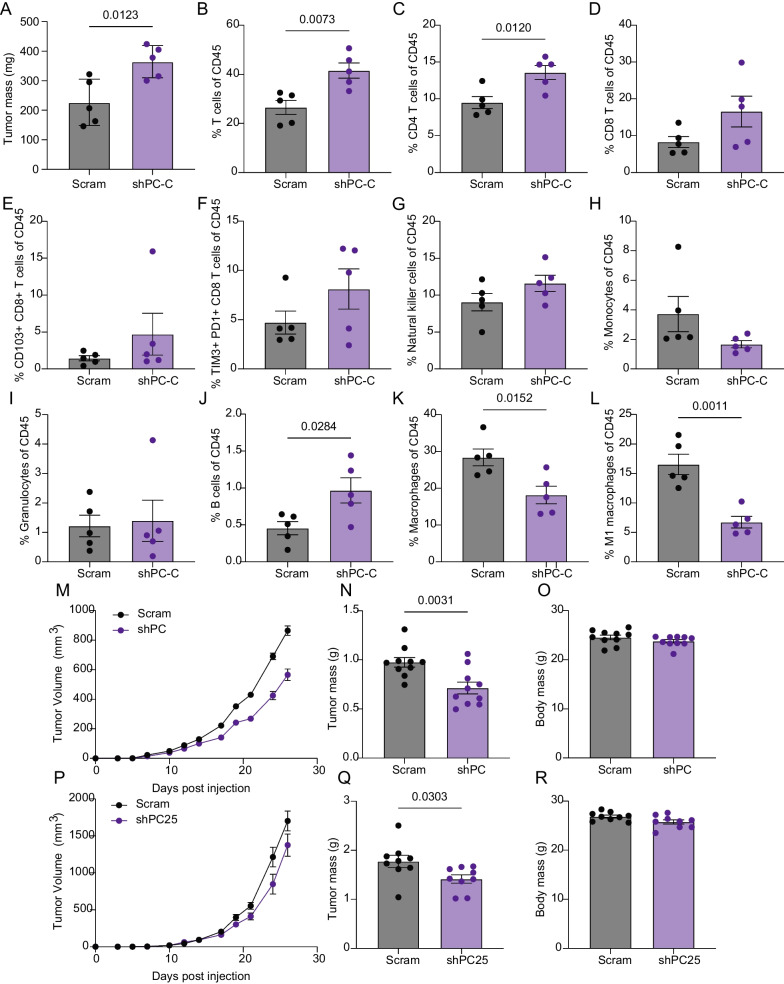
PC depletion remodels tumor immune landscape to promote tumor growth. **A** Tumor mass from M-Wnt tumors harboring control (scram) or PC-targeted (shPC-C) constitutive shRNAs (n = 5/group). **B**–**L** Flow cytometric analysis of CD45+ cells from M-Wnt tumors harboring control (scram) or PC-targeted (ShPC-C) constitutive shRNAs (n = 5/group). Tumor growth over time (**M**), terminal tumor mass (**N**), and terminal body mass (**O**) of NSG mice bearing M-Wnt tumors harboring control (scram) or PC-targeted (shPC-C) constitutive shRNAs (n = 10/group). Tumor growth over time (**P**), terminal tumor mass (**Q**), and terminal body mass (**R**) of NSG mice bearing E0771 tumors harboring control (scram) or PC-targeted (shPC25) constitutive shRNAs (n = 10/group). Statistical significance was determined by unpaired student’s t-test

We used flow cytometry to directly determine whether PC suppression remodels the immune landscape of M-Wnt tumor microenvironment. As anticipated, PC suppression in M-Wnt cells promoted tumor growth (Fig. 3A). Concurrent with accelerated tumor growth, the frequency of T cells, particularly CD4+ T cells, was increased by suppression of PC, without significant changes in either number of or phenotypic state of CD8+ T cells (Fig. 3B–F). Natural killer cells, monocytes, and granulocytes were unchanged, and B cells were more frequent following PC suppression (Fig. 3G–J). Macrophages, and particularly M1 polarized macrophages were increased by PC suppression (Fig. 3K–L). In a separate cohort of mice bearing control and PC-depleted M-Wnt tumors we found that levels of FOXP3+CD4+ T cells increased with PC-depletion (Additional Fig. 3A–C).

Finally, to assess whether changes in immune landscape were required for PC suppression to accelerate tumor growth we orthotopically transplanted scram and shPC E0771 and M-Wnt cells into NSG mice. PC suppression not only failed to accelerate tumor growth but modestly reduced it in NSG mice, reducing both growth over time and terminal tumor mass without altering body weight in M-Wnt (Fig. 3M–O) and E0771 (Fig. 3P–R) tumors.

Overall, these data indicate that unlike the essential role of PC in lung metastasis [24], suppression of PC aids primary tumor development by modulating the tumor immune microenvironment. Indeed, low PC expression is associated with significantly worse survival in patients treated with anti-PDL1 immune checkpoint inhibitors but not anti-PD1 or anti-CTLA4 therapies (Additional Fig. 4A–C). To address the potential for metabolically extrinsic factors to support PC mediated immune evasion we considered whether PC suppression would alter immune checkpoint expression in tumor cells. Depletion of PC resulted in elevated AKT signaling and PDL1 expression, which was abrogated by treatment with the PI3K inhibitor, LY294002 (Additional Fig. 4D–F).

### PC expression is reduced by hypoxia

**Fig. 4:**
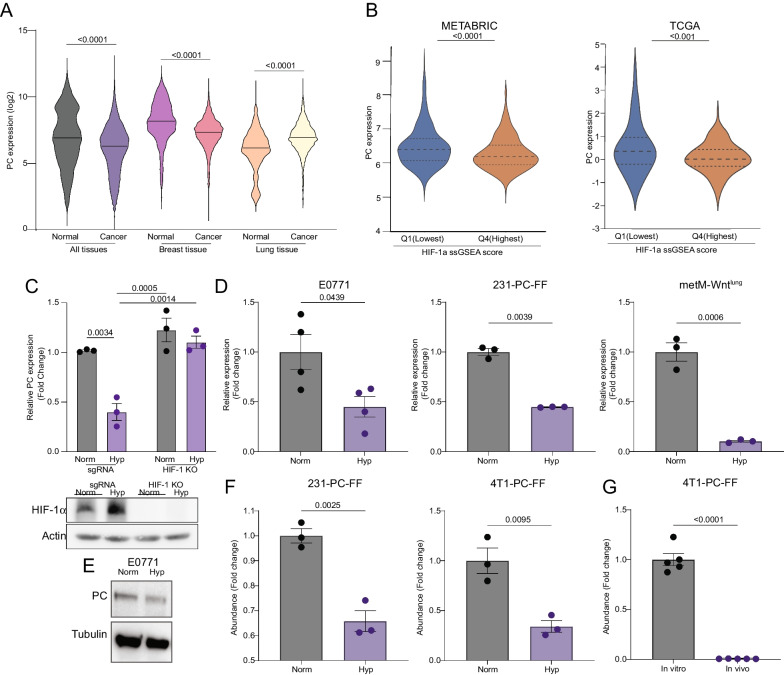
PC expression is reduced by hypoxia. **A** Comparison of PC expression profiles in publicly available databases among all tissues (n = 5487 normal, 35,806 cancer), breast (n = 475 normal, 5555 cancer), and lung tissues (n = 1016 normal, 2316 cancer). **B** PC expression in upper and lower quartiles of HIF-1α signaling ssGSEA score in the METABRIC and TCGA breast cancer databases. **C** PC gene expression determined by qPCR under normoxia (Norm (21% O_2_)) and hypoxia (Hyp (1% O_2_)) in sgRNA control and HIF1ɑ knockout A549 cells (n = 3/group). **D** PC-gene expression determined by qPCR under normoxia (Norm (21% O_2_)) and hypoxia (Hyp (1% O_2_)) in E0771, MDA-MB-231 and metM-Wnt^lung^ cells (n = 3–4/group). **E** PC protein levels in E0771 cells after 48 h of culture in normoxia (Norm (21% O_2_)) and hypoxia (Hyp (1% O_2_)). **F** MDA-MB-231 (231) cells transiently transfected with and 4T1 cells stably expressing firefly luciferase under the control of the PC promoter and renilla luciferase under the control of the CMV promoter cultured under normoxic (Norm, 21% O_2_) and hypoxic (Hyp, (1% O_2_)) conditions and analyzed using the Dual-Glo Luciferase Assay System (n = 3/group). **G** 4T1 cells expressing the PC promoter luciferase reporter described in panel E were orthotopically transplanted for 2 weeks and in vivo PC-firefly/CMV-renilla reporter activity was compared to in vitro cultured cells (n = 5/group). Statistical significance was determined by Mann-Whittney U test (A-B) or unpaired student’s t-test (C-E)

To investigate the prevalence of PC suppression in primary tumors we used data publicly available through GENT2 to compare PC mRNA levels in normal tissues to corresponding primary tumors from patients from either any cancer, or specifically breast or lung cancer. PC expression was significantly lower in primary tumors relative to normal tissue in both the pan-cancer and breast cancer datasets (Fig. 4A). In contrast, PC expression was higher in lung cancer relative to normal (Fig. 4A). Given the key role of PC in directing central carbon metabolism, and the prevalence of hypoxia in solid tumors, we next sought to determine if PC expression was correlated with markers of hypoxia. Gene expression data from patients with breast cancer was obtained from METABRIC and TCGA and stratified into quartiles based on ssGSEA enrichment of HIF-1α signaling and PC expression was determined from RNAseq data.

We found that PC expression was significantly lower in tumors that were enriched for this hypoxia signature (Fig. 4B). Previous studies identified a HIF-1α binding site in the PC proximal promoter [36]. Along these lines, we found that under hypoxic growth conditions, downregulation of PC required expression of HIF-1α (Fig. 4C). Using several models of breast cancer, we found that hypoxic conditions reduced PC at the mRNA and protein levels (Fig. 4D, E). All cell types evaluated demonstrated 50–90% reduction in PC mRNA levels after 24–48-h of hypoxia (Fig. 4D). To further explore the transcriptional regulation of PC, we cloned its proximal promotor, from -800 to + 61 relative to transcriptional start, upstream of firefly luciferase. Hypoxia reduced PC-firefly luciferase reporter activity in MDA-MB-231 and 4T1 cells relative to renilla luciferase driven by a constitutively active CMV promoter (Fig. 4F). We and others have previously demonstrated that orthotopic 4T1 mammary tumors grow at an extreme rate, resulting in these tumors quickly becoming hypoxic [37]. Consistent with their hypoxic nature, our previous studies indicate PC is not detectable by IHC in 4T1 cells when growing as primary tumors [24]. Hence, we tested if growth in such a hypoxic environment might reduce PC promotor activity. Orthotopic tumors generated with 4T1 cells stably expressing the PC-firefly / CMV-Renilla reporters had lower relative PC promoter activity as compared to those same cells cultured under normoxic in vitro conditions (Fig. 4G). Taken together, these data indicate that PC expression is reduced by hypoxic conditions that are characteristic of highly aggressive solid tumors.

### Suppression of PC increases lactate production

**Fig. 5:**
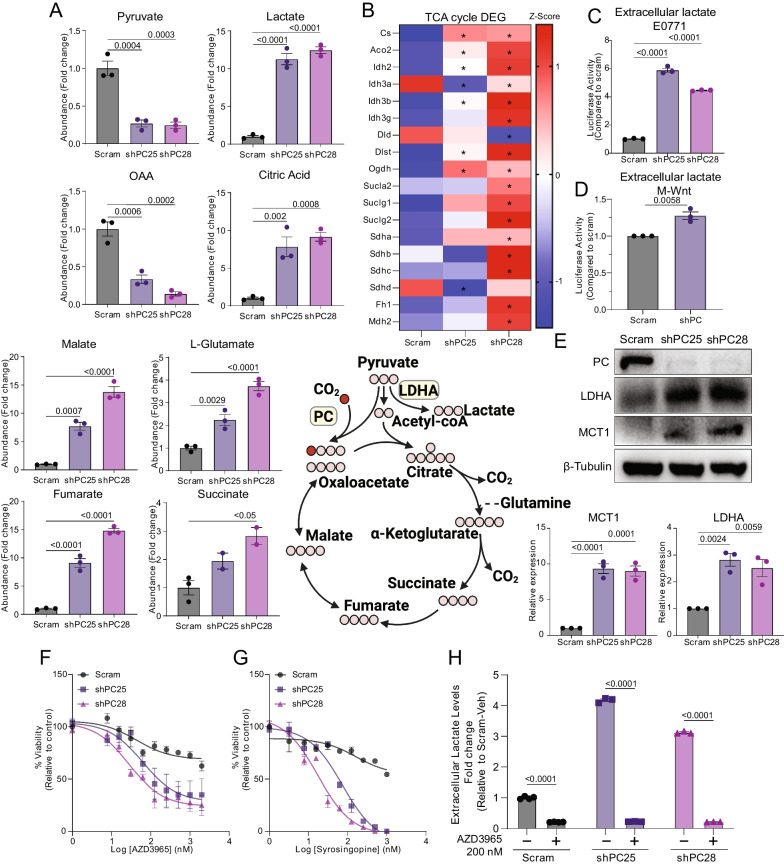
Depletion of PC increases lactate production. **A** Metabolomic screen of glycolytic and TCA cycle intermediates between control and PC-depleted E0771 cells (n = 3 technical replicates /group). **B** Heatmap of differentially expressed TCA cycle genes determined by transcriptomic profiling (FDRq < 0.05) (n = 3/group). **C**, **D** Extracellular lactate concentration in control and PC-depleted E0771 and M-Wnt cells as quantified by luminescence assay. **E** Immunoblot analyses for LDH-A and MCT-1 in control and PC-depleted cells. **F**, **G** Cell viability analyses of control and PC-depleted E0771 cells upon treatment with the MCT-1 inhibitors, AZD3965 (n = 3/group), or the MCT 1 and 4 inhibitor syrosingopine (n = 2/group). **H** Extracellular lactate levels quantified following treatment of control and PC-depleted E0771 cells with 200 nM AZD3965 (n = 3/group). Statistical significance was determined by one-way ANOVA (**A**, **C**, **D**) or two-way ANOVA (F–H) with Tukey's post-hoc tests

We next sought to elucidate potential mechanisms through which repression of PC may promote an immunosuppressed microenvironment. We first conducted metabolomic screening analyses on control and PC-depleted E0771 cells, which revealed that PC regulates levels of both glycolytic and TCA cycle intermediates. As expected, levels of oxaloacetate, the immediate product of PC-mediated carboxylation of pyruvate, were decreased in PC-depleted cells relative to control (Fig. 5A). However, most other TCA cycle intermediates including succinate, fumarate, and malate had increased pool sizes in PC-depleted cells relative to control (Fig. 5A). Further, we observed a tenfold increase in intracellular lactate in PC-depleted E0771 cells as compared with control (Fig. 5A). Concordant with dysregulated TCA cycle metabolism, transcriptomic profiling revealed marked up-regulation of several transcripts encoding TCA enzymes in PC-depleted E0771 cells (Fig. 5B). While some heterogeneity between shRNA constructs was evident, overall directionality of changes relative to control cells was remarkably consistent. We next sought to validate the impact on lactate and thus assayed extracellular lactate levels in PC-depleted cells. Confirming the results of our metabolic screen we found elevated levels of lactate in PC-depleted E0771 and M-Wnt cells relative to controls (Fig. 5C, D). Consistent with increased lactate production and secretion, immunoblot analyses indicated that PC-depleted cells express higher levels of LDH-A and MCT-1 compared with control cells (Fig. 5E). In addition, PC-depleted E0771 cells were more sensitive to the MCT1 inhibitor AZD3965 (Fig. 5F), with similar effects seen with the MCT1/MCT4 dual inhibitor syrosingopine (Fig. 5G). Abrogation of PC-mediated lactate secretion demonstrated the effectiveness of 200 nM AZD3965 to inhibit lactate secretion (Fig. 5H). We also confirmed that depletion of PC increased the sensitivity of M-Wnt cells to the LDHA inhibitor FX-11, and that increased lactate production was reverted by FX-11 treatment (Additional Fig. 6A–C). Finally, we assayed whether PC depletion would alter PDH phosphorylation in normoxia or hypoxia. As expected, in control cells PDH phosphorylation was induced by hypoxia. In contrast, hypoxia was not able to induce PDH phosphorylation in PC-depleted cells (Additional Fig. 6D, E).

**Fig. 6:**
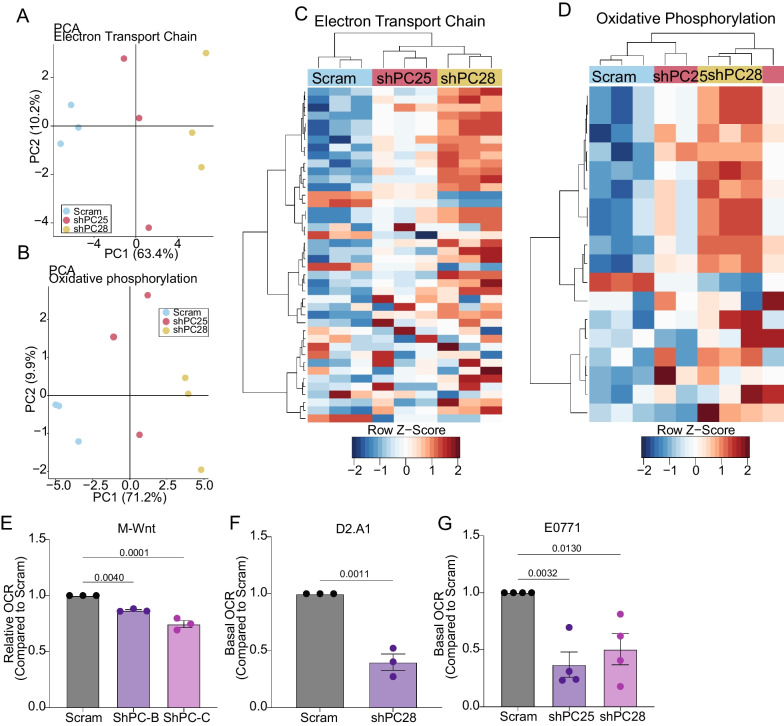
Depletion of PC decreases oxygen consumption. **A**, **B** PCA of gene expression profiles of control (scram) and PC-depleted (shPC25 and shPC28) E0771 cells cultured in low (5.6 mM) glucose DMEM using genes contained in the “electron transport chain” (**A**) and “oxidative phosphorylation” Wikipathways (n = 3/group). **C**, **D** Hierarchical clustering of gene expression profiles obtained under conditions described in panels A and B. **E**–**G** Basal oxygen consumption rates determined by extracellular flux analysis of the indicated control and PC-depleted cells (n = 3–4/group). Statistical significance calculated using one-way ANOVA with Tukey's post-hoc test (**E**, **G**) or unpaired student’s t-test (**F**)

Using transcriptomic profiling of PC-depleted E0771 cells in vitro, we next sought to identify metabolically related pathway alterations. PCA and hierarchical clustering of gene expression, using the member genes of “electron transport chain” or “oxidative phosphorylation” gene lists curated by Wikipathways, demonstrated distinct clustering of cell lines (Fig. 6A–D). To characterize metabolic differences between control and PC-depleted cells, we next performed extracellular flux analyses. PC suppression in three different mammary cancer cell lines decreased oxygen consumption rate (OCR) compared with their PC-expressing counterparts (Fig. 6E–G).

To determine if the reduction in OCR observed in PC-depleted cells was due to electron transport chain intrinsic defects, we used high resolution respirometry to measure mitochondrial OCR in the presence of non-limiting ETC substrates. Reduced O_2_ consumption was not found with any combination of complex I and II substrates tested following suppression of PC in M-Wnt cells. Indeed, shPC-B showed an increased in complex I and II mediated O_2_ consumption (Additional Fig. 6A). Similarly, when O_2_ consumption for each sample at each stage in the SUIT protocol were normalized to peak O_2_ consumption (PMGS_E_), no alterations in flux control ratio were observed (Additional Fig. 6B). Together, these data suggest that suppression of PC impairs TCA metabolism and oxidative phosphorylation independent of electron transport chain intrinsic defects.

Finally, we asked whether forced overexpression of PC in 4T1 cells would complement the phenotype of PC suppression observed in M-Wnt and E0771 cells. In vitro, overexpression of PC in 4T1 cells suppressed LDHA expression and decreased lactate production without altering basal OCR or ECAR (Additional Fig. 7A–D). In vivo*,* overexpression of PC in promoted primary tumor growth, but reduced pulmonary metastasis (Additional Fig. 7E–G). In primary tumors, PC-overexpression increased Ki67, reduced CD4, and did not alter CD8-positive cells per field (Additional Fig. 7H–J). In contrast, lung metastases from PC overexpressing tumors did not have altered Ki67, but increased CD4 and CD8-positive cells per field (Additional Fig. 7K–M).

### Immunosuppression following depletion of PC requires lactate secretion

**Fig. 7:**
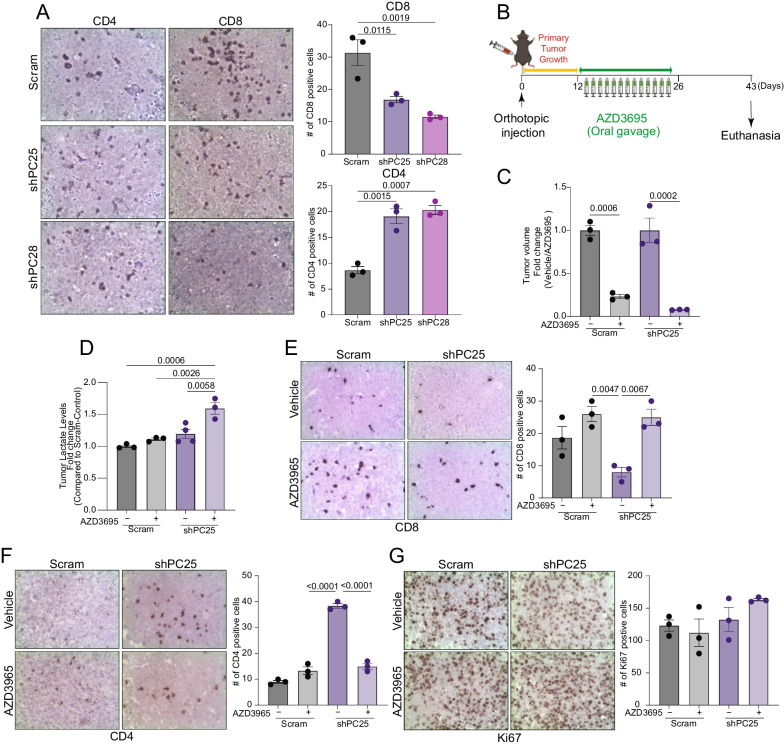
Depletion of PC modulates the tumor immune microenvironment. **A** (left panel) Representative IHC staining of sections from control and PC-depleted E0771 tumors. (Right panel) Quantification of CD8^+^ and CD4^+^ T cell numbers (n = 3/group). **B** Experimental summary of the in vivo study. Mice were orthotopically engrafted with control and PC-depleted E0771 cells (5 × 10^5^ cells per mouse) and their primary mammary tumor growth was observed for 10 days at which point AZD3965 was administered. **C** Fold change of tumor volume in response to AZD3965 (n = 3/group). **D** Lactate levels in the indicated tumors determined by luminescent assay (n = 3–4/group). **E**–**G** (left panels) Representative IHC for CD8, CD4, and Ki67 staining of tumor sections from control and PC-depleted E0771 tumors under control and AZD3965 treated conditions. (Right panel) Quantification of staining CD8^+^ and CD4^+^ T cells, and Ki67^+^ cells (n = 3/group). Statistical significance determined by one-way ANOVA with Tukey's post-hoc test

Given our observed increases in lactate production and secretion upon PC depletion in Fig. 5 we next sought to determine if suppressing PC alters the abundance of tumoral CD4^+^ and CD8^+^ T cells. We quantified CD4^+^ and CD8^+^ lymphocytes in tumors from control and PC-depleted groups by immunohistochemistry. Consistent with our gene expression analyses, we observed lower numbers of CD8^+^ lymphocytes and higher numbers of CD4^+^ T cells in PC-depleted E0771 tumors (Fig. 7A). To determine if extracellular lactate mediates intratumoral lymphocyte abundance, we treated both control and PC-depleted tumor bearing mice with the MCT-1 inhibitor, AZD3965, every day for two weeks (Fig. 7B). MCT-1 inhibitor treatment significantly reduced tumor growth in both control and PC-depleted groups, but significant accumulation of intracellular lactate was only observed in the PC-depleted tumors (Fig. 7C, D).

Importantly, immunohistochemical analyses of those tumor samples clearly demonstrated that MCT-1 inhibition restored CD4^+^ and CD8^+^ lymphocyte abundance in PC-depleted tumors (Fig. 7E, F). Finally, MCT-1 inhibition did not alter the number of Ki67 positive cells in the tumors of any group, indicating that the antitumor effect of MCT-1 inhibition was not explained by global reduction of proliferation (Fig. 7G). These results demonstrate that the modulation of the tumor immune microenvironment by PC-depletion is dependent on lactate secretion.

## Discussion

Cancer cell metastasis and immunosuppression both rely on remodeled tumor cell metabolism [3, 4]. We and others have shown that PC supports the metabolic demands for developing tumors [23–25, 38–41]. In contrast, herein we describe a model whereby expression of PC is transcriptionally suppressed by hypoxia. Under these conditions, diminished levels of PC support immunosuppression via increased production of extracellular lactate (graphical abstract). Concordant with our findings, recent work has identified suppression of PC in TAMs to limit antitumor immunity, with both hypoxia and soluble factors from the TME required for PC suppression [26]. Similarly, PC expression in CD8+ T cells is essential to support effective activation and antitumor immunity [42]. While the exact mechanism of how hypoxia reduces PC promoter activity is yet to be determined, our study builds a framework for understanding the role of PC in mammary tumors as a key hypoxia-responsive factor that drives metabolic reprogramming and immunosuppression.

Hypoxia-mediated changes in tumor metabolism remain incompletely understood [43]. Hypoxia limits oxidative phosphorylation via activation of pyruvate dehydrogenase kinase (PDK) [44] and promote lactate metabolism by direct activation of lactate dehydrogenase (LDH) enzymes [45, 46] and MCT transporters [47]. Our work extends these findings by demonstrating that under hypoxic conditions, PC is transcriptionally repressed via its proximal promoter, and loss of PC promotes increased lactate production. These findings are consistent with patient data that indicate PC expression is lower in many solid tumors relative to normal tissue. Further, PC expression is inversely associated with a hypoxic signature of primary breast tumors. Moreover, our previous studies also indicate that the extremely hypoxic primary tumors resulting from mammary fat pad engraftment of 4T1 cells also lack PC expression [24].

To recapitulate the loss of PC expression in 4T1 mammary tumors, we utilized various shRNA constructs to deplete PC in the E0771 and M-Wnt cells, as they spontaneously metastasize at lower rates than 4T1 cells. Indeed, unlike our previous studies in the 4T1 model [24], specific depletion of PC in both E0771 and M-Wnt cells increased primary tumor growth. Complementing our depletion approach, we demonstrate that overexpression of PC in the 4T1 cells correspondingly led to decreased lactate production and dramatic immune attack of establishing metastases. These data suggest that hypoxia-mediated suppression of PC in primary tumors contributes to immune evasion as cells disseminate. However, data from our group and others clearly indicate that at some point in establishment of macrometastasis, return of PC expression is required. Therefore, the intricacies of the return of PC expression upon reoxygenation and the contribution of cellular heterogeneity in PC expression to metastatic initiation and outgrowth need to be further explored.

Metabolomic analyses revealed that PC suppression leads to the expected depletion of oxaloacetate and increased levels of citric acid. Interestingly, under nutrient replete culture conditions we also observed increased levels of glutamate, succinate, fumarate, and malate. These data suggest increased dependency on glutamine in the absence of PC, and are consistent with previous reports that indicate higher levels of pyruvate and lower levels of glutamine drive the requirement of PC-dependent anaplerosis in the pulmonary microenvironment [25]. In contrast, glutamine levels dominate in primary mammary tumors, potentially explaining why PC expression is dispensable in our studies.

Lactic acidosis is a clinical consequence of genetic PC deficiency in newborns [48]. These data provide strong clinical precedent for PC downregulation leading to increased lactate production. Here, we demonstrate that PC-depleted cells produce more lactate and are more sensitive to inhibition of lactate metabolism using compounds targeting lactate synthesis and secretion. Taken together these data suggest that PC suppression in primary tumors may drive increased glutamine utilization for anaplerotic metabolism to support rapid lactate efflux.

Suppression of PC also reduced markers of tumor immunosurveillance in both tumor models used in this study. IHC staining further supported these gene expression differences by demonstrating reductions of CD8^+^ T cells and increases of CD4^+^ T cells in PC-depleted tumors relative to controls. The finding that loss of PC in cancer cells regulates CD8^+^ and CD4^+^ T cell populations in tumors further advances our understanding of lactate-mediated suppression of antitumor immunity [6, 10, 16]. Our finding that pharmacological inhibition (via AZD3965) lactate transport reversed remodeling of tumoral T cell populations is striking as it indicates that not only was loss of PC expression sufficient to promote these effects, but also highlights lactate metabolism as an exploitable dependency.

## Conclusions

Taken together, our findings link tumor hypoxia to aberrant production of lactate via suppression of PC. In contrast to the essential role of PC for metastatic progression within the oxygen rich microenvironment of the lungs, the current study presents an important relationship between hypoxia, metabolic plasticity, and regulation of tumor immunity. This work expands a growing body of evidence that supports PC as an important regulator of both antitumor immunity and cancer metabolism.

### Supplementary Information

Additional file 1 (PDF 130 kb)

Additional file 2 (PDF 162 kb)

Additional file 3 (PDF 106 kb)

Additional file 4 (PDF 206 kb)

Additional file 5 (PDF 389 kb)

Additional file 6 (PDF 157 kb)

Additional file 7 (PDF 346 kb)

Additional file 8 (DOCX 14 kb)

Additional file9 (DOCX 14 kb)

## Data Availability

The transcriptomic datasets supporting the conclusions of this article are available in the GEO database under accession GSE199831 (https://www.ncbi.nlm.nih.gov/geo/query/acc.cgi?acc=GSE199831). All other data is contained within the article and its additional files, further details available on request to the corresponding authors (SDH and MKW).

## Abbreviations

*PC*: Pyruvate carboxylase
*LDHA*: Lactate dehydrogenase
TME: Tumor microenvironment
MCT: Monocarboxylate transporter
OAA: Oxaloacetate
TCA: Tricarboxylic acid
PDK: Pyruvate dehydrogenase kinase
