# Supplementary figures and images for: Hypoxia-mediated repression of pyruvate carboxylase drives immunosuppression
Source: Breast Cancer Res. 2024 Jun 7;26:96. doi: 10.1186/s13058-024-01854-1 (PMC11161980; doi:10.1186/s13058-024-01854-1)

# Additional figure 1

## M-Wnt cells

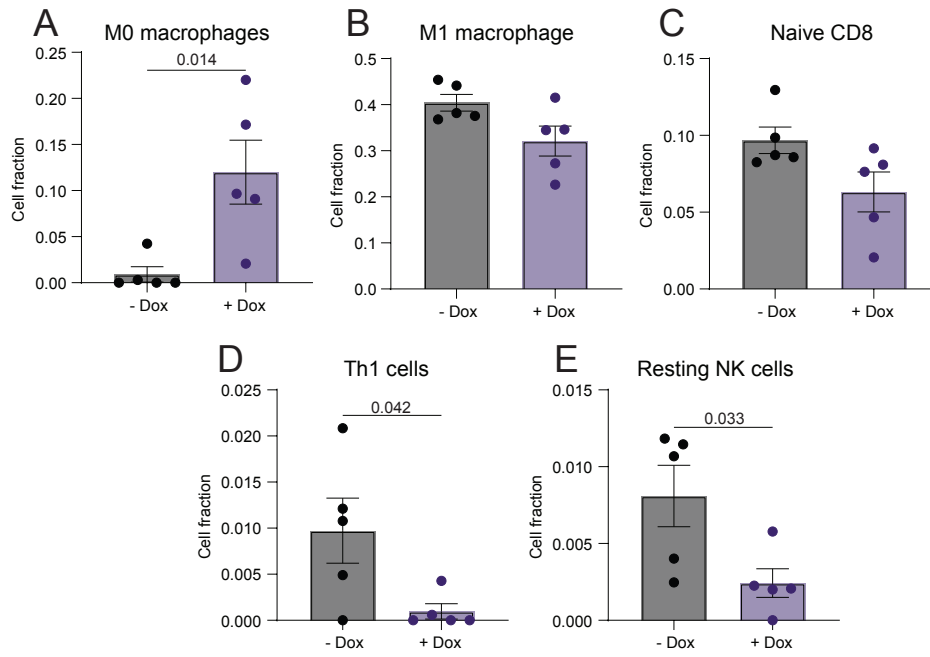

Supplement: Supplementary file 1 — Additional file 1 (PDF 130 kb) [file 13058_2024_1854_MOESM1_ESM.pdf]

## Additional figure 2

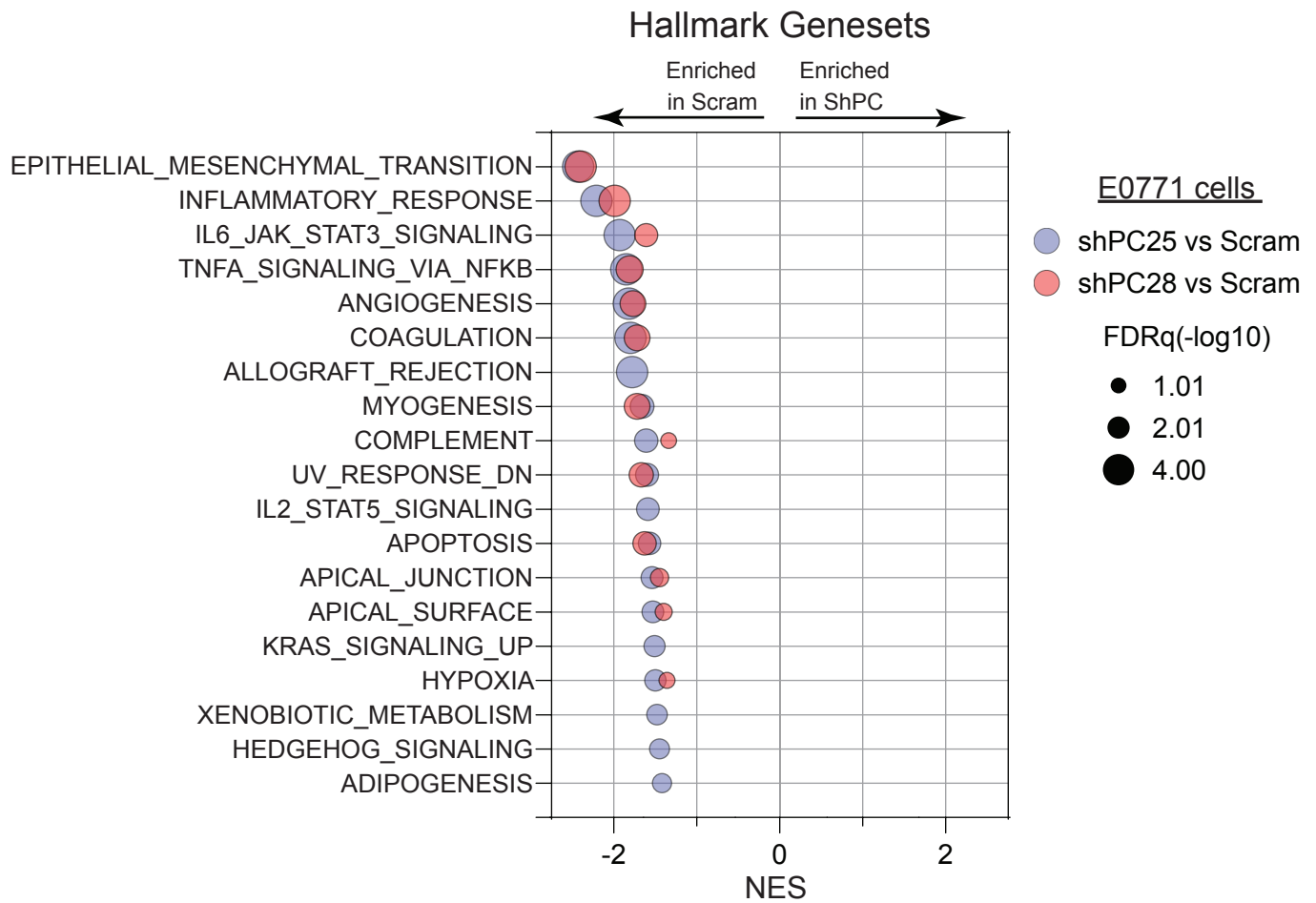

Supplement: Supplementary file 2 — Additional file 2 (PDF 162 kb) [file 13058_2024_1854_MOESM2_ESM.pdf]

Additional Figure 3

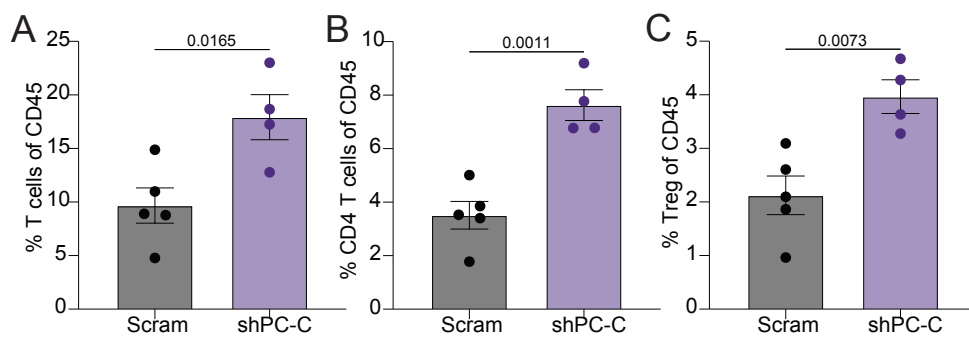

Supplement: Supplementary file 3 — Additional file 3 (PDF 106 kb) [file 13058_2024_1854_MOESM3_ESM.pdf]

# Additional figure 4

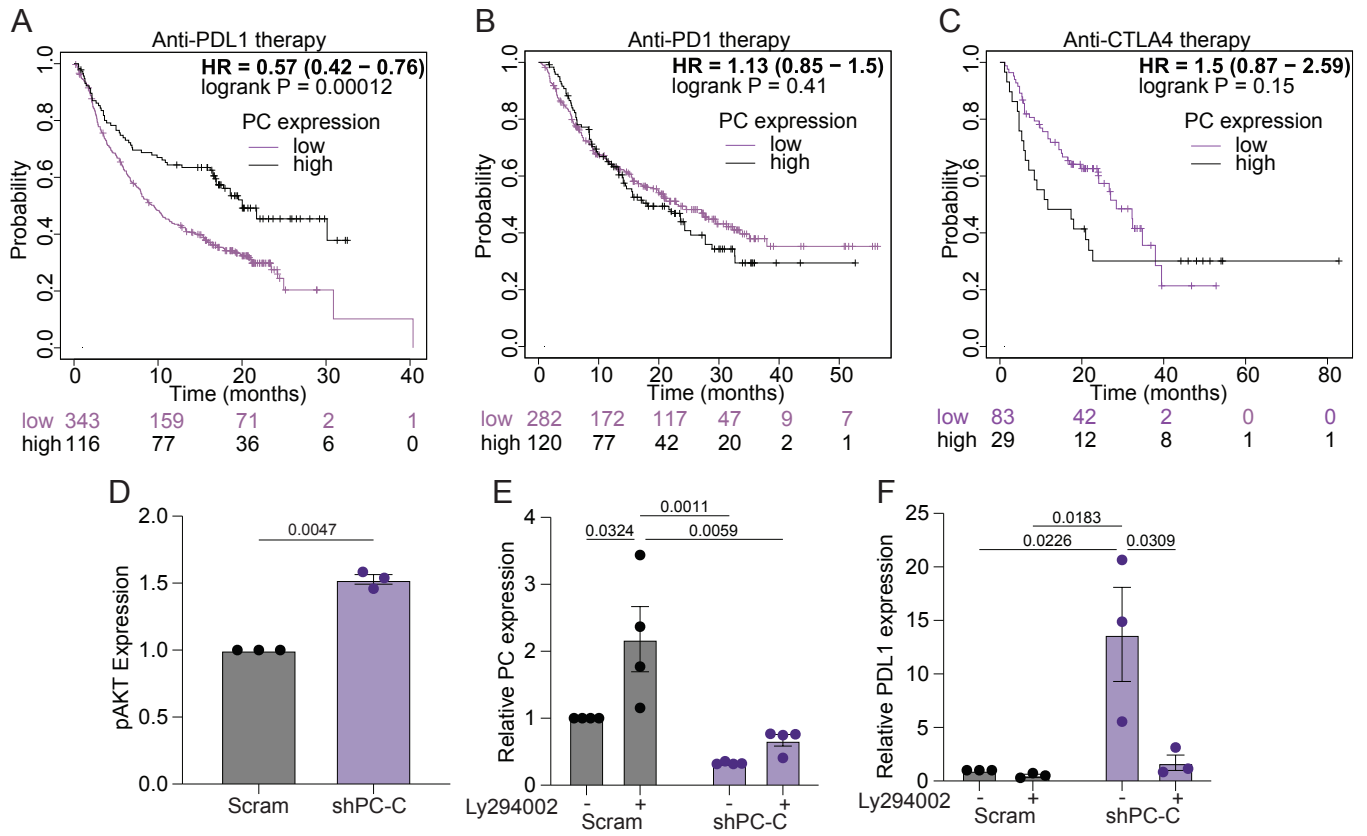

Supplement: Supplementary file 4 — Additional file 4 (PDF 206 kb) [file 13058_2024_1854_MOESM4_ESM.pdf]

# Additional figure 5

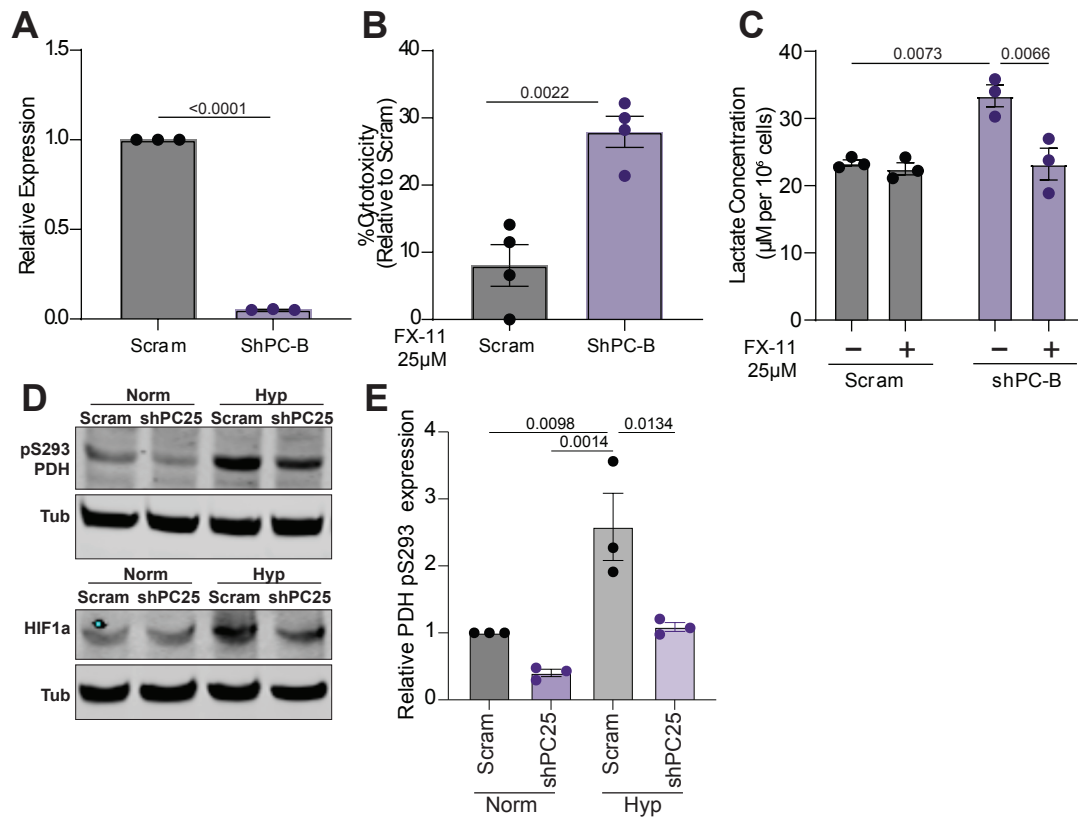

Supplement: Supplementary file 5 — Additional file 5 (PDF 389 kb) [file 13058_2024_1854_MOESM5_ESM.pdf]

Additional figure 6

A

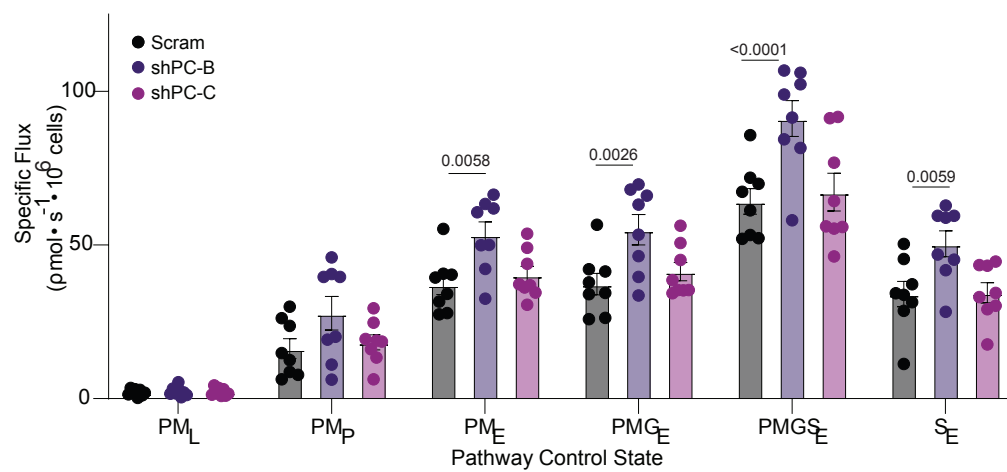

B

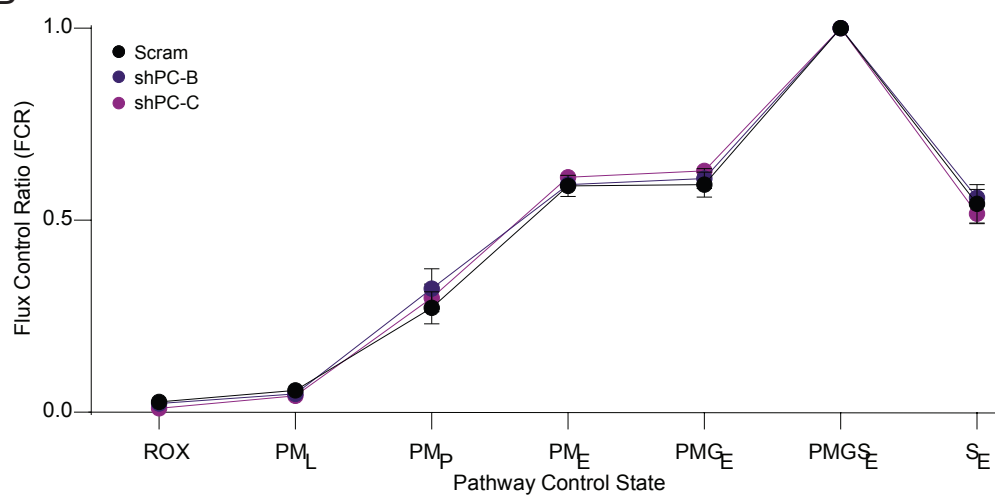

Supplement: Supplementary file 6 — Additional file 6 (PDF 157 kb) [file 13058_2024_1854_MOESM6_ESM.pdf]

# Additional figure 7

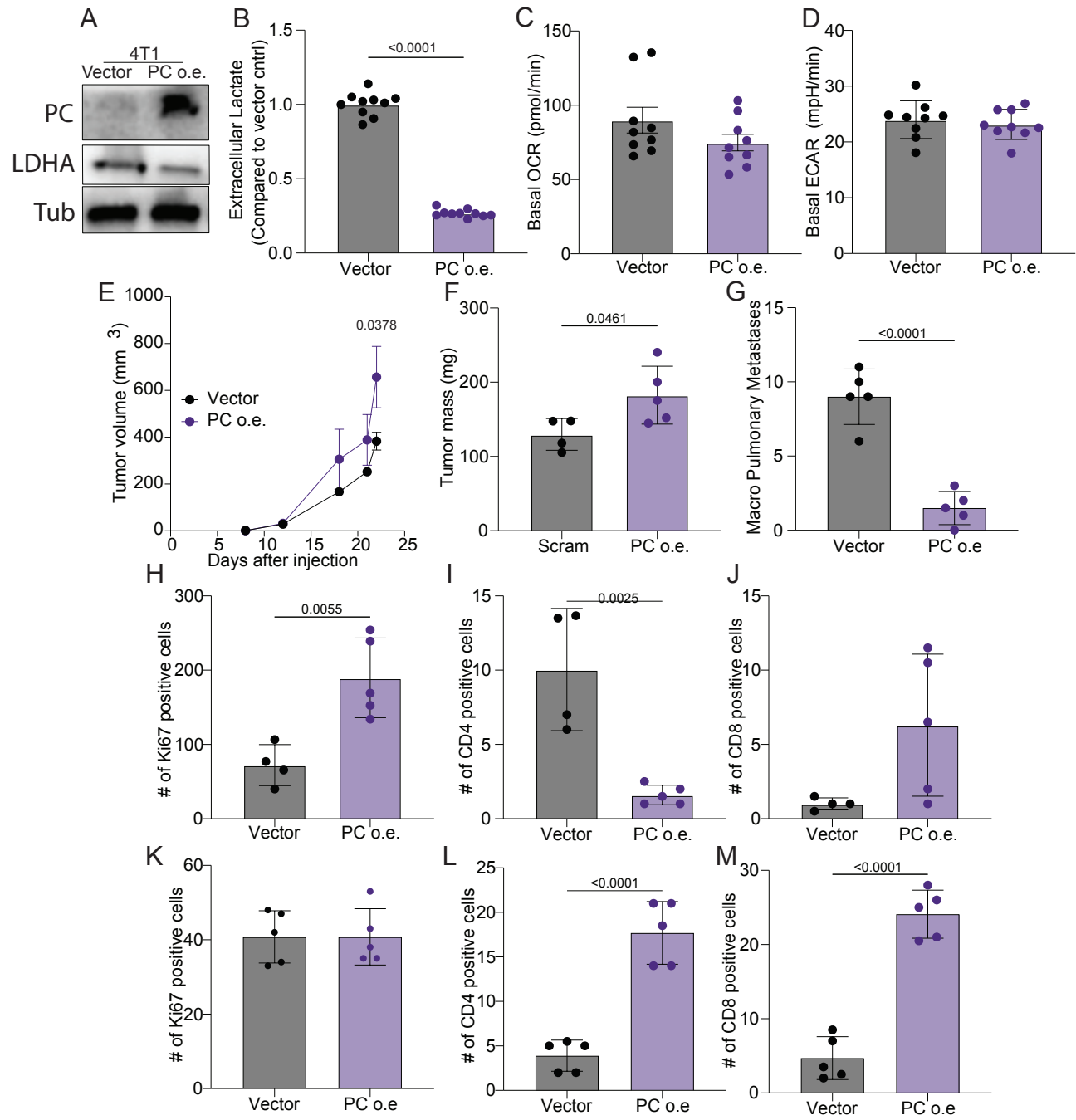

Supplement: Supplementary file 7 — Additional file 7 (PDF 346 kb) [file 13058_2024_1854_MOESM7_ESM.pdf]
