# Supplementary material for: Hypoxia-mediated repression of pyruvate carboxylase drives immunosuppression
Source: Breast Cancer Res. 2024 Jun 7;26:96. doi: 10.1186/s13058-024-01854-1 (PMC11161980; doi:10.1186/s13058-024-01854-1)
Supplement: Supplementary file 8 — Additional file 8 (DOCX 14 kb) [file 13058_2024_1854_MOESM8_ESM.docx]

**Additional Figure 1: Digital cytometry of + Dox and - Dox tumors**

Digital cytometry based on transcriptomic profiler from control (- Dox) and PC suppressed (+ Dox) M-Wnt tumors (n=5/group). (A) M0 Macrophages, (B) M1 Macrophages, (C) naïve CD8 T cells, (D) TH1 cells, and (E) resting NK cells (n=5/group). Statistical significance was determined by Student’s t-test (A-E). All graphs represent mean ± SEM.

**Additional Figure 2: Suppression of PC in E0771 tumors suppresses immune related gene expression**

Hallmark GSEA of transcriptomic profile of tumors from control and PC-suppressed E0771 tumors, significantly enriched with FRDq <0.05 (n=3/group).

**Additional Figure 3: Suppression of PC in M-Wnt tumors promotes Treg infiltration**

(A-C) Flow cytometric analysis of CD45+ cells from M-Wnt tumors harboring control (Scram) or PC-targeted (ShPC-C) constitutive shRNAs (n=4-5/group). Statistical significance was determined by Student’s t-test.

**Additional Figure 4: Hypoxia signature is associated with low PC expression**

PC expression in and upper and lower quartiles of HIF-1α signaling ssGSEA score in the TCGA breast cancer database.

**Additional Figure 5: Suppression of PC in predicts response to immunotherapy and induces PDL1 expression.**

Overall survival analysis of patients treated with (A) anti-PDL1 (n=459), (B) anti-PD1(n=402), or (C) anti-CTLA4 (n=112) immunotherapies stratified by PC expression using KMPlotter automatic expression threshold. (D) pAKT S473 expression in PC suppressed (shPC-C) or control (Scram) M-Wnt cells (n=3/group). (E) PC and (F) PDL1expression in PC suppressed (shPC-C) or control (Scram) M-Wnt cells treated with or without 20nM LY294002 (n=4/group). Statistical significance was determined by Logrank test (A-C), Student’s t-test (D), and two-way ANOVA (E-F).

**Additional Figure 6: Suppression of PC increases reliance on lactate production independent of PDH phosphorylation**

(A) Expression of PC in control (scram) and PC-suppressed (ShPC-B) M-Wnt cells was quantified by qPCR (n=3/group). (B) Cell viability analysis of control and ShPC-B cells upon treatment of 25𝜇M FX-11(n=4/group). (C) Intracellular lactate concentration in control and ShPC-B cells as quantified by using luminescent assay(n=3/group). (D-E) Immunoblot analysis of Scram and shPC25 E0771 cells cultured for 24 h in normoxia (21%O_2_) or hypoxia (1%O_2_) and probed with indicated antibodies. Statistical significance determined by Student’s t-test (A-B) and one-way ANOVA (C,E).

**Additional Figure 7: PC suppression does not promote electron transport chain intrinsic defects**

High resolution respirometry of permeabilized M-Wnt cells. (A) Specific oxygen flux of control (scram) and PC-suppressed (ShPC-B and ShPC-C) M-Wnt cells (𝜌mol∙s-1∙million cells) (n=8/group). (B) Flux control ratio (FCR) normalized to the internal reference state PMGSE, achieved following addition of N- and S-coupled substrates and titration of FCCP until complete uncoupling of the mitochondrial membrane potential was achieved. Statistical significance determined by one-way ANOVA.

**Additional Figure 8: Overexpression of PC in 4T1 cells suppresses lactate production and promotes tumor growth**

(A) PC overexpression in 4T1 cells. (B) Extracellular lactate levels in PC overexpressed (PC o.e.) or vector control (Vector) 4T1 cells (n=10/group). (C-D) Basal OCR and ECAR in PC overexpressed (PC o.e.) or vector control (Vector) 4T1 cells (n=9/group). PC overexpressed (PC o.e.) or vector control (Vector) 4T1 cells were orthotopically translated into BALB/c mice and (E) tumor growth over time, (F) ex vivo tumor mass, and (G) pulmonary metastasis determined (n=5/group). (H-M) Quantification of Immunohistochemistry staining of Ki67, CD4, and CD8 in Vector and PC o.e. primary tumors (H-J) and pulmonary metastasis (K-M) (n=4-5/group). Statistical significance determined by Students t test (B-D, G-M) or two-way ANOVA (E).
