# Supplementary material for: Hypoxia-mediated repression of pyruvate carboxylase drives immunosuppression
Source: Breast Cancer Res. 2024 Jun 7;26:96. doi: 10.1186/s13058-024-01854-1 (PMC11161980; doi:10.1186/s13058-024-01854-1)
Supplement: Supplementary file 9 — Additional file9 (DOCX 14 kb) [file 13058_2024_1854_MOESM9_ESM.docx]

| **Target** | **Species** | **Forward** | **Reverse** |
| --- | --- | --- | --- |
| PC | Mouse | TCA CCA GTG ACT CTG TCA AAC | GAC CAG GTC CAC ATC TGT AAT C |
| PC | Human | ATG TTG CCC ACA ACT TCA GCA AGC | AGT TGA GGG AGT CAA ACA CAC GGA |
| GAPDH | Mouse | CAA CTT TGG CAT TGT GGA AGG GCT C | GCA GGG ATG ATG TCT TGG GCA GC |
| GAPDH | Human | TGC ACC ACC AAC TGC TTA GC | GGC ATG GAC TGT GGT CAT GAG |
| PDL1 | Mouse | GCT CCA AAG GAC TTG TAC GTG | TGA TCT GAA GGG CAG CATT TC |
| RPLP0 | Mouse | AGA TTC GGG ATA TGC TGT TGG C | TCG GGT CCT AGA CCA GTG TTC |
| UBC | Mouse | CTG GAA GAT GGT CGT ACC CTG | GGT CTT GCC AGT GAG TGT CT |

**Table S1 Oligonucleotide primer sequences**
